# Hyperbaric Oxygen Therapy to Treat Diabetes Impaired Wound Healing in Rats

**DOI:** 10.1371/journal.pone.0108533

**Published:** 2014-10-15

**Authors:** Bastiaan Tuk, Miao Tong, Esther M. G. Fijneman, Johan W. van Neck

**Affiliations:** Department of Plastic and Reconstructive Surgery, Erasmus MC, University Medical Center, Rotterdam, the Netherlands; Hungarian Academy of Sciences, Hungary

## Abstract

Wound healing in diabetes is frequently impaired and its treatment remains a challenge. Hyperbaric oxygen therapy (HBOT) receives a wide attendance and is often used as a last resort treatment option, however, its effectiveness for many conditions is unproven. We tested the effect of HBOT on healing of diabetic ulcers in an animal experimental setting. Experimental diabetes was induced by intraperitoneal injection of streptozotocin. Four weeks after diabetes induction, rats were ulcerated by clamping a pair of magnet disks on the dorsal skin for 16 h. After magnet removal, the animals received HBOT, daily on weekdays, for 4 weeks. To examine the effect of HBOT on diabetes impaired wound healing, the degree of wound tissue perfusion, inflammation, angiogenesis, and tissue breaking strength were evaluated. HBOT effects on the degree of inflammation and number of blood vessels could not be observed. HBOT improved the tissue breaking strength of the wound, however, this did not reach statistical significance. Twenty hours after ending the HBOT, a significantly improved oxygen saturation of the hemoglobin at the venous end of the capillaries and the quantity of hemoglobin in the micro-blood vessels was measured.

## Introduction

Wound healing failure in diabetes represents a significant clinical problem with an predicted further increase in patient numbers and impact [1]. The pathophysiology of diabetic ulcers is largely due to hyperglycemia and implicates blood vessel abnormalities and damage, particularly in the microvasculature, that may lead to the low oxygen tension causing tissue hypoxia and ischemia [2]. In patients with diabetes, this underlying pathology is considered to affect foot ulcer chronicity. The impaired healing observed in diabetic patients and diabetic animal models, overlap in many aspects, such as a disturbed inflammatory response, an abnormal microvasculature and an aberrant collagen synthesis and ripening [3].

The medical use of hyperbaric oxygen (hyperbaric oxygen therapy: HBOT) is the use of pure oxygen at an increased pressure. Frequently used is a treatment regimen where the patient breathes 100% oxygen at a pressure of 2.4 atmospheres absolute (ATA) for 1.5–2 hours, on a daily basis during weekdays, for 6 to 8 weeks. During treatment, the arterial oxygen tension is described to rise over 2000 mmHg, and levels of 200 to 400 mmHg occur in tissues in part due to an increased plasma oxygen content and microvascular blood flow [4]. In a The Cochrane Collaboration Review on Hyperbaric oxygen therapy for chronic wounds, Kranke et al (2012) concluded that HBOT may improve the chance of healing of diabetes-related foot ulcers and may reduce the number of major amputations in these patients [5]. Due to obvious reasons, efficacy determination for the use of HBOT in clinical studies does not allow the in depth laboratory analysis that can be reached when using animal models.

Therefore, in this study, the effect of HBOT on improving oxygenation of the wound bed was analysed in a preclinical setting using a validated ischemia experimental wound model in streptozotocin (STZ)-diabetic rats, with an approximately two-fold delay in ulcer healing time [3].

## Materials and Methods

### Animals

WAG/RijHsd female rats (n = 24, 8 weeks old) were purchased from Charles River (l'Arbresle, France). The rats were exposed to a 12-hour light/dark cycle and fed with a standard laboratory diet (Hope Farms, Woerden, The Netherlands) with food and water available ad libitum. The rats were allowed to acclimatize to their environment for one week prior to the procedure. The experimental protocol was approved by the Animal Experiments Committee(Permit Number EMC2585) under the national experiments on animals act and adhered to the rules laid down in this national law that serves the implementation of “Guidelines on the protection of experimental animals” by the Council of Europe (1986), Directive 86/609/EC.

### Induction of diabetes

After overnight fasting, animals were given an intraperitoneal injection of STZ (Sigma-Aldrich, St. Louis, MO) at a dose of 60 mg/kg body weight in 0.05 mol/L sodium citrate buffer, pH 4.5. Blood glucose concentration was monitored twice weekly the first 3 weeks and weekly afterwards by a OneTouch glucometer (LifeScan, Milpitas, CA) from tail vein blood. Glucose levels of 33 mmol/L and above were displayed by the apparatus as 33 mmol/L. A prolonged diabetes status was defined as blood glucose levels ≥20 mmol/L throughout the entire diabetes induction period.

### Ulceration model and HBOT

Four weeks after STZ injection, two rats returned to normal glucose levels and were excluded from further experimentation. Twenty two diabetic rats had a prolonged diabetic status and were ulcerated by clamping a pair of magnet disks (15-mm in diameter) on the dorsal skin for a single ischemic period of 16 h. This procedure gave drop in perfusion during clamping to at least 40% of the starting perfusion and a recovery perfusion of 60% and resulted in 2 wounds per animal [3], [6]. The rats received temgesic 0,05 mg/kg (Reckitt Benkiser Pharmaceuticals, Berkshire, UK) during magnet clamping and three days post-operative. Immediately after magnet removal, rats were randomly allocated into two groups to serve the two experimental end points (i.e., day 7, n = 6; day 29, n = 16). The rats in each group were randomly assigned to the HBOT or normoxia group. HBOT rats were given 100% oxygen under a pressure of 2.4 ATA for 90 min. The control group received normoxia at sea level pressure. During HBOT, animals were single housed in cages [7]. Control rats were placed next to the machine to experience analogous stress created by the noise of the machinery and breathed normoxia at normal pressure. The first HBOT was initiated approximately 30 min after the magnet was removed and continued once a day from Mondays through Fridays until the experimental end point was reached and the animals were euthanized. For all experimental outcome, observers were blinded to the treatment.

### Macroscopic analysis

Body weight and blood glucose levels were measured and the ulcers were photographed, all on a weekly basis.

### Perfusion measurements

Oxygen supply in the microcirculation of the wound tissue was measured at the wound edges using an O2C laser doppler flow meter and tissue spectrometer (LEA Medizintechnik, Giessen, Germany). Capillary-venous oxygen saturation of the hemoglobin (SO2) was measured at the venous end of the capillaries, which represents the lowest oxygen saturation of the tissue. The relative amount of hemoglobin (rHB) represents the quantity of hemoglobin in the micro-blood vessels and, therefore, reflects the density of the blood vessels. In addition, the blood flow in the microcirculation was determined [8]. Intra- and inter-operator variability and measurement probe repositioning was secured and the sensitivity of the measurement determined by measuring an animal directly after HBOT. We could demonstrate high SO2 values that gradually lowered in time. The results of some of these measurements can be found in Data S2.

### Immunohistochemistry

At the experimental endpoint at day 7, the entire wound was paraffin-embedded. At experimental endpoint at day 29, the wound area was cut into two halves. One half was paraffin embedded. The other half was used to measure the breaking strength. Paraffin-embedded sections (5 µm), n = 6 per group and time point, were deparaffinized and rehydrated. Antigen retrieval was performed in Tris-EDTA buffer containing 0.1% trypsin (Invitrogen, Carlsbad, CA). Endogenous peroxidase activity was quenched by exposing to 0.1% hydrogen peroxide in PBS containing 0.1% Tween 20 (PBST). After blocking with 4% nonfat milk powder in PBST, the sections were incubated with mouse anti-CD68 (1∶100; AbD Serotec, Düsseldorf, Germany) and goat anti-CD34 (1∶200; DakoCytomation, Glostrup, Denmark), respectively, followed by incubating with the corresponding biotinylated secondary antibodies (DakoCytomation). The antigen-antibody complex was detected by streptavidin-peroxidase (DakoCytomation) and 3,3′-diaminobenzidine (Sigma-Aldrich, Zwijndrecht, the Netherlands).

### Breaking strength measurements

At experimental day 29, the wound area was cut into two halves. One half was paraffin embedded. The other half was used to measure the breaking strength as described previously [9]. In brief, the excised dorsal pelt which contained the ulcer, was cut into two standardized dumbbell-shaped skin strips. One strip was cut from the non-wounded skin surrounding the wound area and the other strip was centered by the mid segment of the ulcer. The strip was fixed perpendicularly between two clips of a tensiometer (Testometric AX, M250-2.5KN, Testometric Company Ltd., Lancashire, UK) and subjected to a constant strain rate of 60 mm/min using a 10-kg force transducer. Breaking strength was recorded as the maximum load (in Newton) measured before skin failure. The ratio of ulcer breaking strength to that of surrounding normal skin breaking strength was calculated for data analysis. In control animals at experimental day 29, the breaking strength of non-wounded skin was determined in 7 skin strips (1 strip per animal). the breaking strength of wounded skin in 14 skin strips (1 strip per wound, 2 wounds per animal). In HBOT animals at experimental day 29, the breaking strength of non-wounded skin was determined in 9 skin strips (1 strip per animal). The breaking strength of wounded skin in 18 skin strips (1 strip per wound, 2 wounds per animal).

### Statistical analysis

Data are presented as means ± SEM. Statistical calculations were performed using IBM SPSS software, version 21 (Chicago, IL). An independent samples Student’s t test was carried out to compare results between groups. P-values ≤0.05 were considered to indicate statistically significant differences.

## Results

### Diabetes induction

During the 3.5–4 week diabetes induction period, 22 STZ-injected rats became consistently hyperglycemic, with glucose levels >20 mmol/L during the entire experimental period, and were included in this study. Two rats returned to normoglycemia and were excluded from the experiment (Table 1).

**Table 1:** Group data for blood glucose (mmol/L) for the control group and HBOT group.

blood glucose at:	HBOT group	control group
diabetes induction	5.3±0.2	6.5±0.4
wound induction	32.4±0.4	33.0±0
week 1	32.6±0.4	33.0±0
week 2	32.1±0.5	33.0±0
week 3	32.1±0.6	32.7±0.3
week 4	32.2±0.4	32.4±0.6

Data are presented as means ± SEM. A glycometer reading of 33 represents tail vein glucose levels of 33 mmol/L and above.

### Bodyweight changes

During the 3.5–4 weeks of diabetes induction, the biggest individual weight loss observed was 15%. On a per group bases, the biggest weight loss was 10% for animals in the control group and 4% for animals in the HBOT group (statistically non significant: NS). At the end of the diabetes induction period, all animals increased weight and almost returned to their starting weight 140±2 gram versus 134±2 gram: NS, for HBOT-group and control-group animals, respectively). During the experimental period, also no significant differences in animal weight were observed between the control and HBOT group (Table 2).

**Table 2:** Group data for body weight (gram) for the control group and HBOT group.

Animal weight percentage at	HBOT group	control group
diabetes induction	146±3	140±2
wound induction	140±2	134±2
week 1	144±2	136±2
week 2	143±2	140±4
week 3	141±3	139±3
week 4	139±2	136±3

Data are presented as means ± SEM.

### Histological observations

**Figure 1:**
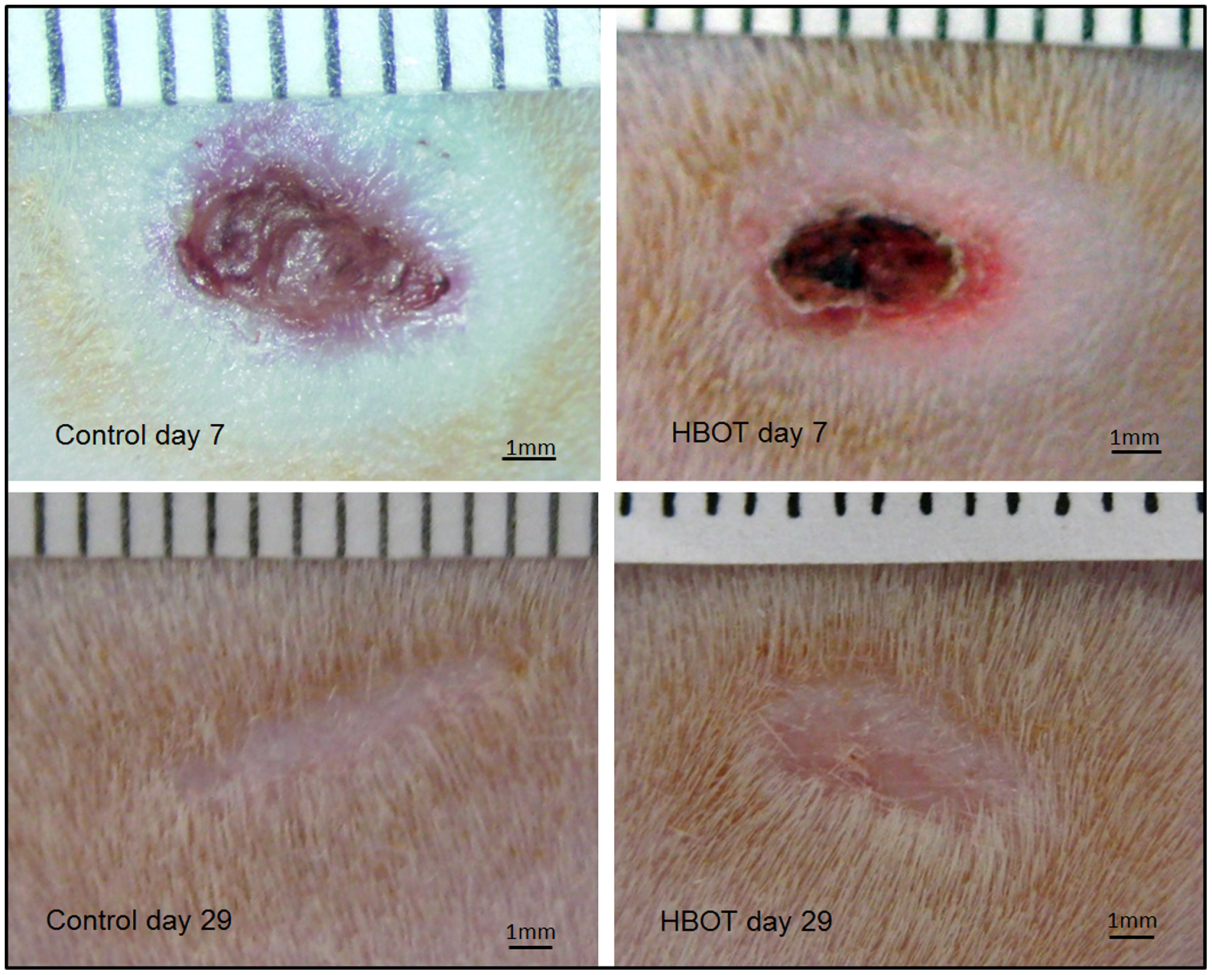
Macroscopic images of control and HBOT wounds at post-wounding days 7 and 29.

**Figure 2:**
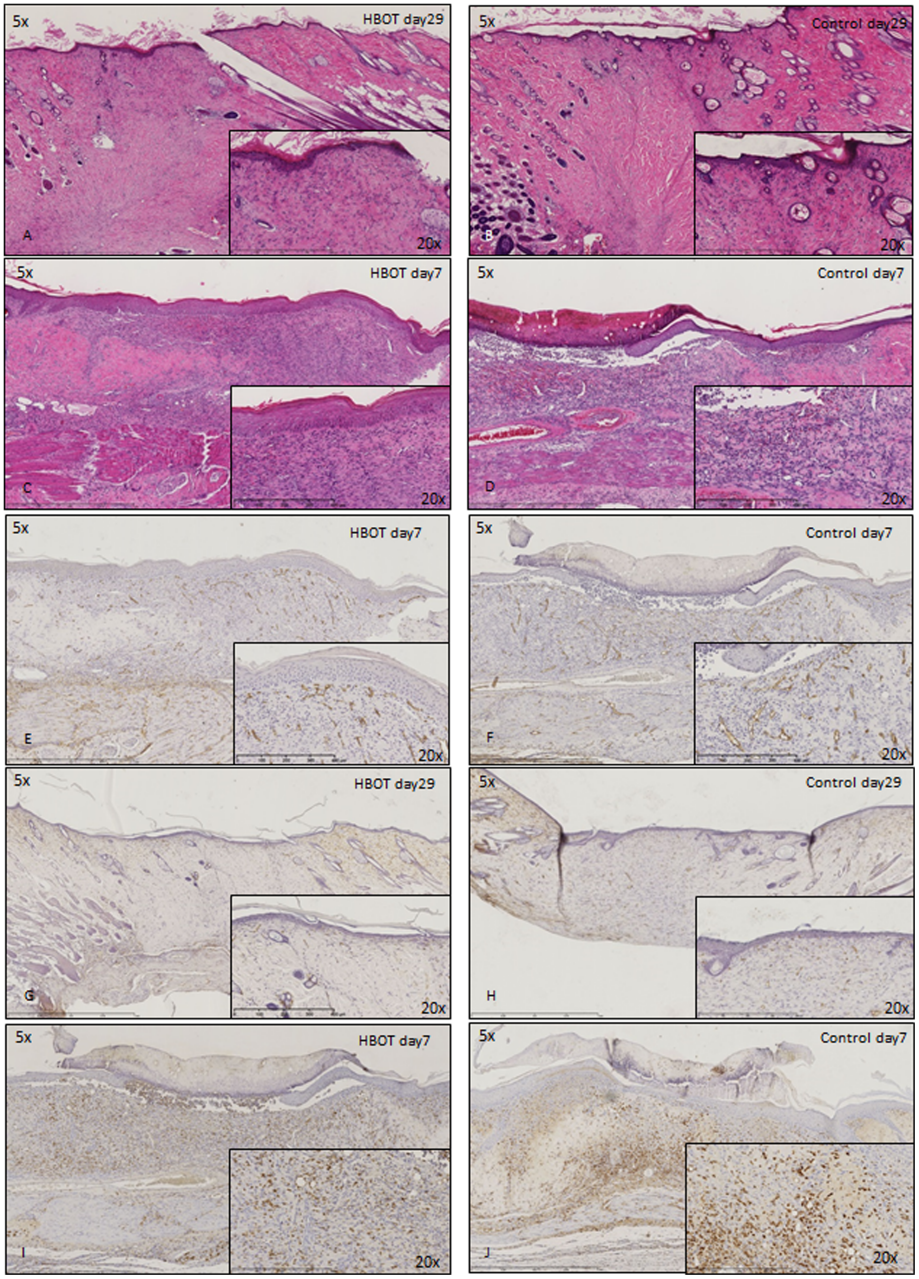
Histological staining of control and HBOT wounds at post-wounding days 7 and 29. A–D) H&E staining. E–H) CD34 immunohistochemistry. I+J) CD68 immunohistochemistry.

Both at experimental days 7 and 29, no differences between the control and HBOT group were observed in the macroscopic observation of the wound area (Figure 1). Also in an overall histological view of the wound (H&E stain) (Figure 2A–D) and in the number of blood vessels (CD34 immunohistochemistry) (Figure 2E–H) or macrophages (CD68 immunohistochemistry) (Figure 2I, J) no obvious differences were observed.

### Perfusion measurements

Approximately 20 hours after the last HBOT (or control) session at experimental day 29, the capillary-venous oxygen saturation (SO2), the relative amount of hemoglobin (rHB) and blood flow in the microcirculation were determined in non-wounded (control) skin and at the wound edge.

**Figure 3:**
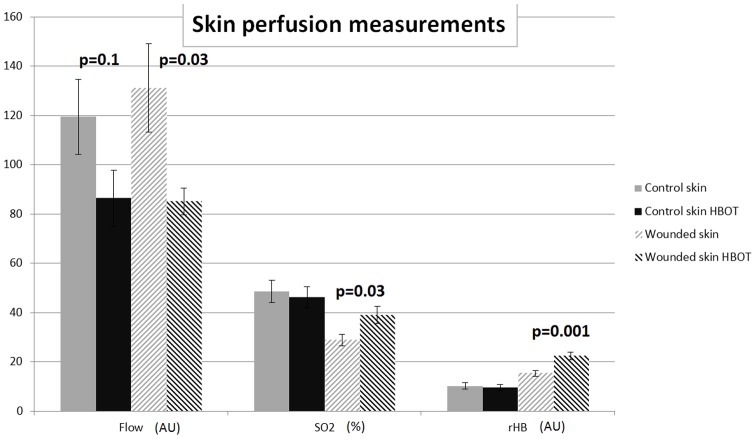
The effect of HBOT on blood flow, oxygen saturation of the hemoglobin at the venous end of the capillaries (SO2), and the quantity of hemoglobin in the micro-blood vessels (rHB). Effects were measured, in control skin and at the wound edge in control and HBOT animals, approximately 20 hours after the last HBOT session. Data are presented as means ± SEM. P-values indicate differences between the respective control group and HBOT group. AU = artificial units.

In control skin, a trend towards a reduced flow was found (119.5±15.2 versus 86.4±11.4 artificial units (AU): p = 0.1, for control and HBOT animals, respectively). This reduction in flow reached significance in wound tissue (131.2±18.0 versus 85.1±5.3 AU: p = 0.03, for control and HBOT animals, respectively) (Figure 3). Oxygen saturation of the hemoglobin (SO2) at the venous end of the capillaries was not significantly changed in control skin, however, was significantly increased in HBOT wound tissue (28.9±2.4% and 39.1±3.5%: p = 0.03, for control and HBOT animals, respectively). Also the relative hemoglobin (rHB), the quantity of hemoglobin in the micro-blood vessels, is significantly increased in HBOT wound tissue (15.4±1.2 versus 22.4±1.5 AU: p = 0.001, for control and HBOT animals, respectively) (Figure 3).

**Figure 4:**
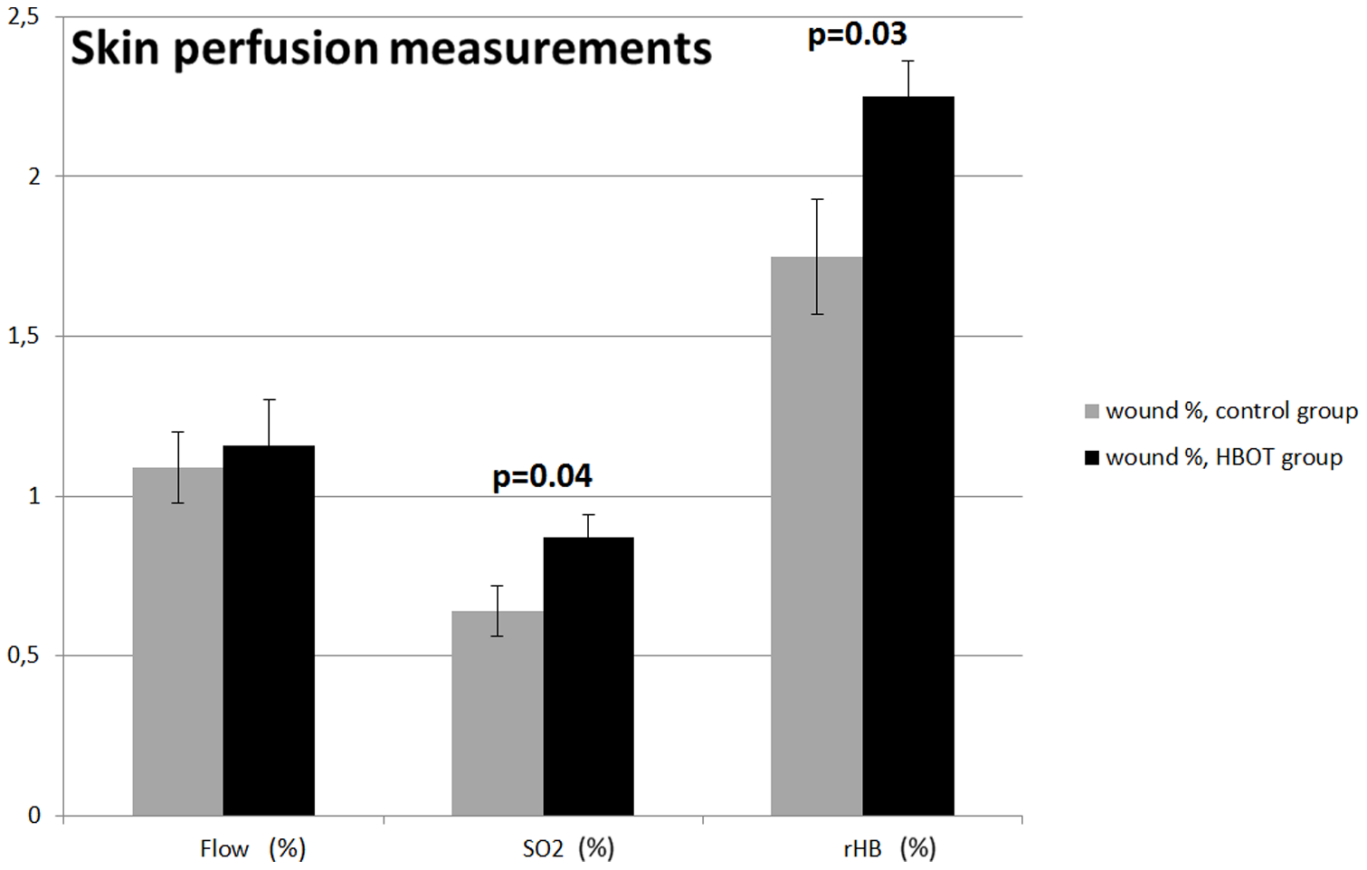
The effect of HBOT on blood flow, oxygen saturation of the hemoglobin at the venous end of the capillaries (SO2), and the quantity of hemoglobin in the micro blood vessels (rHB). Effects were measured in control and HBOT animals, approximately 20 hours after the last HBOT session, and expressed as a percentage of the values of non-wounded skin in the respective animal. Data are presented as means ± SEM. P-values indicate differences between the respective control group and the HBOT group.

In order to compensate for possible animal to animal variation in tissue architecture and vascular characteristics, we recalculated the above wounded skin perfusion parameters as a percentage of the skin perfusion of the normal skin in the respective animal. In this case, flow in the microcirculation did not reach statistical difference (1.09±0.11 versus 1.16±0.14: NS, for control and HBOT animals, respectively) indicating that HBOT restricts blood flow both in control and wounded skin. In contrast, oxygen saturation of the hemoglobin at the venous end of the capillary system (SO2∶0.64±0.08 versus 0.87±0.07, p = 0.04, for control and HBOT animals, respectively) and the quantity of hemoglobin in the micro-blood vessels (rHB: 1.75±0.18 versus 2.25±0.11: p = 0.03, for control and HBOT, respectively) where increased in the HBOT group (Figure 4).

### Skin breaking strength measurements

**Figure 5:**
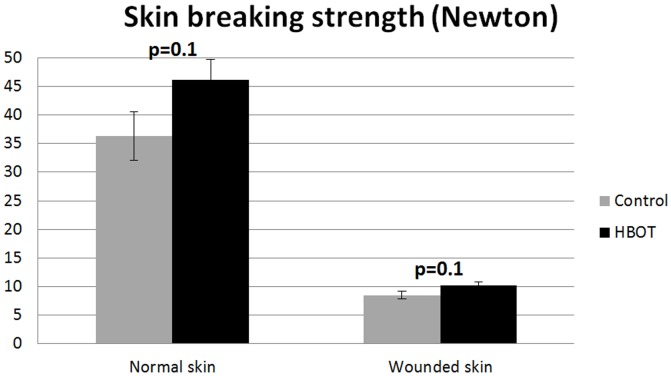
Ratio of the skin breaking strength of normal skin and wounded skin in diabetic rats at day 29 post wounding. Data are presented as means ± SEM. P-values indicate differences between the control group and the HBOT group.

At the experimental endpoint at day 29, HBOT treatment improved the mean breaking strength of unwounded skin by 27%, compared to controls, however, this did not reach statistical significance (36.29±4.29N versus 46.16±3.57N: p = 0.1, for control and HBOT, respectively). Also the mean skin strength ratio in HBOT-treated ulcers was improved by 19% but this observation did not reach statistical significance (8.51±0.70N versus 10.16±0.65N: p = 0.1, for control and HBOT, respectively) (Figure 5).

## Discussion

This study demonstrated effects of 4 weeks of HBOT on the skin of diabetic animals. HBOT significantly improved oxygen saturation and the quantity of hemoglobin in the micro blood vessels in the wound area. HBOT also improved the tissue breaking strength of the wounded and non-wounded skin of these diabetic rats but this did not reach statistical significance.

In this STZ-diabetic rat model, glucose levels and animal weights were stable during the entire diabetes induction and experimental period. HBOT treatment did not influence this. HBOT animals displayed a significant reduction in blood flow in the wound area. In contrast, oxygen saturation of the hemoglobin at the venous end of the capillaries (SO2), and the quantity of hemoglobin in the micro-vessels (rHB) both were significantly increased in the wounded skin of HBOT treated animals. Since we measured tissue perfusion 20 hours after the final HBOT treatment, this reflects a lasting effect of HBOT in oxygenating the wound area. Klemetti et al. also reported a lasting increase in vascular capacity following HBOT [10]. In their rat mandible bone defect model, increased Laser Doppler Flow values in the wound were observed 14 days after the HBOT ended.

The mechanism of action of HBOT is considered to occur via increasing the oxygen content of poorly vascularized areas. A number of HBOT effects are described in literature some having conflicting predictions on wound healing. Vasoconstriction is a well described effect of HBOT [11], likely mediated by the inactivation of nitric oxide through superoxide radicals [12]. We also observed a HBOT reduced blood flow of which a negative impact to wound healing can be envisioned. In contrast, a variety of additional HBOT effects exist that likely have a positive result on wound healing. We observed an increase in hemoglobin oxygenation and perfusion. In addition, the administration of pure oxygen at a raised pressure of 2.4 ATA, is thought to give oxygen dissolved plasma levels that are sufficient for the tissue, even without the support of HB-bound oxygen. This way, plasma exvasation is considered to increase the oxygen content of lesser vascularized areas [11], as in poorly vascularized wounds [13]. Fife et al [14] demonstrated, in humans with diabetic lower extremity wounds, the direct relationship between transcutaneous oxygen tension (TcPO2) measurements during HBOT and healing improvement of this treatment.

In addition to this direct effect on oxygen availability in tissue, it is suggested that HBOT promotes angiogenesis during wound healing via an increased availability of wound growth factors, of which VEGF is most noted [15]. Sheikh et al. demonstrated increased VEGF levels in wound fluid aspirates of surgical wounds in rats that were treated with HBOT twice daily [16]. VEGF levels returned to normal three days after ending HBOT. Increased VEGF, and enhanced angiogenesis, mostly is described in reaction to hypoxia conditions [17]. All together, these data may indicate that increased VEGF levels arise as a response to deviation from normoxia. Yuan et al. applied HBOT to *in vitro* aortic preparations and assayed the NO and VEGF production [18]. No direct effect of HBOT on VEGF expression was noted. However, a mild increase of VEGF, following HBOT, was observed when the culture medium was supplemented with lactate to mimic the poorly oxygenated wound conditions.

Although skin breaking strength (failure force) was improved by about 20% in the HBOT animals, this observation did not research statistical significance. This HBOT effect was observed in wounded skin but also in normal (non-wounded) diabetic skin revealing a general improvement of the skin perfusion by HBOT. Compared to the skin of normal animals, diabetic skin has a significantly reduced epidermal thickness and is less vascularized (our unpublished findings). In a previous study, we reported on the effects of a glycosaminoglycan mimic, OTR4120, on improving diabetic wound repair [3]. Here, wound breaking strength was increased 50% but limited to the wound area. The breaking strength of the unwounded skin was not improved suggesting the absence of prolonged matrix damage in this diabetes model. However, the increase in skin breaking strength in wounded and non-wounded skin of the HBOT group may reflect an improvement in dermal architecture. However, in a histological analysis, no evidence regarding alteration of the skin architecture or in the number of blood vessels in the skin, could be observed in the HBOT groups. Nevertheless, subtle differences in skin architecture like collagen cross linking and/or differences in collagen organization may underlay this observation and may be below the threshold of a histological observation.

When we compare our current findings on the speed of wound healing, the superior oxygen saturation and quantity of haemoglobin observed with HBOT, did not reveal striking differences when compared to our previous study where were tested the effect of matrix therapy on wound healing [3]. However, a disclaimer needs to be made as, in the current study we followed wound healing for only 4 weeks in contrast to 12 weeks in the study of Tong [3].

An experimental limitation of our study is that we only could measure oxygenation characteristics linked to the capillary blood vessels. Direct extravascular oxygenation of the wound bed was not determined. A limitation of our ischemia wound model is that it is a delayed wound healing model. Therefore, we are unable to study the effects of HBOT on healing arrested chronic wounds as observed in some patients. Nevertheless, the study has its merit as the healing arrested chronic wounds also may enter the healing phase during HBOT. Limitations of our experimental design are that non-diabetic control animals are lacking and that perfusion measurements only are taken 20 hours after the last hyperbaric treatment. Therefore, it is unknown if the observed HBOT effects on tissue perfusion are due to the entire course of treatments or only to the last treatment.

In our study we complied the clinical HBOT protocol recommendation consisting of treatments on 20–40 workings days during 90–120 minutes at a pressure of 2.4 atmospheres absolute. A deviation from this protocol, however, is that humans normally are placed in a pressurized air chamber and breathe pure oxygen through a mask for 20–30 minutes, alternated by a short period of normoxia. This approach is considered to limit the toxic side effects of hyperoxia [15]. As our animals do not tolerate breathing through a mask, the HBO chamber that was used for this study is completely filled with pressurized pure oxygen. Thereby omitting the normoxia periods. To our knowledge, effects of these normoxia periods on the effectiveness of HBOT in improving wound healing are not described in literature.

In summary, we observe a lasting improved tissue oxygenation by HBOT in our diabetic rat wound model. Effects on improving the breaking strength and the healing of the wounded skin are in need of further research.

## Supporting Information

Data S1 **Raw data file.**
(XLS)

Data S2 **Repeatability and reliability of the O2C apparatus.**
(XLS)
